# Consulting communities on feedback of genetic findings in international health research: sharing sickle cell disease and carrier information in coastal Kenya

**DOI:** 10.1186/1472-6939-14-41

**Published:** 2013-10-14

**Authors:** Vicki Marsh, Francis Kombe, Raymond Fitzpatrick, Thomas N Williams, Michael Parker, Sassy Molyneux

**Affiliations:** 1 Kenya Medical Research Institute (KEMRI) Wellcome Trust Research Programme, PO Box 230, Kilifi 80108, Kenya; 2 Centre for Tropical Medicine, Nuffield Department of Medicine, Oxford University, Churchill Hospital, Old Road, Oxford OX3 7LT, UK; 3 Ethox Centre, Nuffield Department of Population Health, Oxford University, Rosemary Rue Building, Old Road Campus, Old Road, Oxford OX3 7LF, UK; 4 Nuffield Department of Population Health, Oxford University, Rosemary Rue Building, Old Road Campus, Old Road, Oxford OX3 7LF, UK; 5 Department of Medicine, Imperial College, St Mary’s Hospital, London W21NY, UK

**Keywords:** Kenya, Africa, Sickle cell disease, Community consultation, Genetic findings, Genetic and genomics research, Deliberative methods, Empirical ethics

## Abstract

**Background:**

International health research in malaria-endemic settings may include screening for sickle cell disease, given the relationship between this important genetic condition and resistance to malaria, generating questions about whether and how findings should be disclosed. The literature on disclosing genetic findings in the context of research highlights the role of community consultation in understanding and balancing ethically important issues from participants’ perspectives, including social forms of benefit and harm, and the influence of access to care. To inform research practice locally, and contribute to policy more widely, this study aimed to explore the views of local residents in Kilifi County in coastal Kenya on how researchers should manage study-generated information on sickle cell disease and carrier status.

**Methods:**

Between June 2010 and July 2011, we consulted 62 purposively selected Kilifi residents on how researchers should manage study-generated sickle cell disease findings. Methods drew on a series of deliberative informed small group discussions. Data were analysed thematically, using charts, to describe participants’ perceptions of the importance of disclosing findings, including reasoning, difference and underlying values. Themes were derived from the underlying research questions and from issues emerging from discussions. Data interpretation drew on relevant areas of social science and bioethics literature.

**Results:**

Perceived health and social benefits generated strong support for disclosing findings on sickle cell disease, but the balance of social benefits and harms was less clear for sickle cell trait. Many forms of health and social benefits and harms of information-sharing were identified, with important underlying values related to family interests and the importance of openness. The influence of micro and macro level contextual features and prioritization of values led to marked diversity of opinion.

**Conclusions:**

The approach demonstrates a high ethical importance in many malaria endemic low-to-middle income country settings of disclosing sickle cell disease findings generated during research, alongside provision of effective care and locally-informed counselling. Since these services are central to the benefits of disclosure, health researchers whose studies include screening for sickle cell disease should actively promote the development of health policy and services for this condition in situations of unmet need, including through the prior development of collaborative partnerships with government health managers and providers. Community consultation can importantly enrich ethical debate on research practice where in-depth exploration of informed views and the potential for difference are taken into account.

## Background

Sickle cell (SC) disease is a serious single gene disorder common in many malaria endemic parts of Africa; areas that account for three quarters of an estimated 300,000 to 500,000 children born with SC disease worldwide every year [1]. The high prevalence stems from an evolutionary link between the SC gene and resistance to malaria, a feature that also underpins the common inclusion of SC screening in health research in malaria endemic settings where the gene may act as a risk factor. For example, in the setting for this paper in Kilifi, where around 1% children under one year of age have SC disease and 18% carry SC trait, assessment of SC status has been included in descriptive and intervention studies on malaria, pneumonia, Human Immunodeficiency Virus/Acquired Immunodeficiency Syndrome (HIV/AIDS) and malnutrition in young children as well as in studies on SC disease. An ethical question may then arise about the importance of sharing findings on sickle cell status generated during studies; an issue paradigmatic of more general debates in the literature on researchers’ responsibilities for disclosing study-generated genetic findings with participants, including the additional challenges presented where services for health problems related to findings are not widely available.

SC disease, an autosomal recessive condition, is an inherited abnormality of red blood cells. Affected children inherit two copies of an abnormal haemoglobin gene, one from each parent. For couples where both individuals carry one copy of the abnormal gene, described as having SC trait or being a carrier for SC disease, there is a 1 in 4 chance of future children being affected by the disease. From a biomedical perspective, a high potential for benefit from sharing research-generated SC disease findings stems from a positive health impact of comprehensive forms of health care. Without care, symptoms can be very severe and life threatening, mainly resulting from obstruction to small blood vessels, chronic anaemia, acute breakdown of blood cells and increased risk of serious infection [2,3]. Although environmental and genetic factors influence severity, without care many children in malaria endemic settings are likely to die in their first few years of life [4,5]. In contrast, quality of life is significantly improved where comprehensive care programmes are in place, typically in high-income settings [6,7], leading to a median adult survival of 48 years [8]. SC trait is generally seen as a benign condition [9,10] whose main implication is an increased future reproductive risk for the disease [11].

From the literature, key features of the debate on the general importance of sharing study-generated genetic information with participants are the benefits to participants in practice, including social benefits such as knowing about future reproductive risks [12]; the need to include a wide range of views in understanding the nature and importance of potential benefits [13]; and the validity of testing processes. Over time, consensus in guidelines on disclosure has moved towards recommending greater openness [13], mainly drawing on ethical principles of respect for autonomy of, and maximising benefits for, participants [12]. For SC trait, in addition to generating participants’ awareness of future reproductive risks, an importance has been seen in alerting the wider family to this risk, and respecting their rights to ownership of genetic information [14]. Challenges to disclosure of SC disease findings arise where services needed to generate benefits (such as specialist knowledge, clinical care and counselling) are not available [15]; and, for both disease and trait, by the risks of research being seen as a form of clinical service and issues of research resource prioritisation [12]. Additional risks for SC trait findings are that the health implications will be misinterpreted, stigmatisation and - for children screened - of undermining their autonomy [16]. While the potential for benefit in sharing study-generated SC findings is likely to be closely linked to the presence of effective health services for the condition, these are not widely available in many malaria-endemic settings, with some localised exceptions [17,18]. The potential benefits of sharing study-generated SC disease findings may then depend on forms of ancillary care provided by researchers [19].

Against this background, and building on on-going community engagement activities within the research programme [20,21], this paper reports on a study aiming to inform research practice through exploring the views of a diverse group of Kilifi residents on how researchers should manage study-generated information on sickle cell disease and carrier status. Community engagement has been seen as essential to supporting ethical conduct of research, particularly for international collaborative research conducted in low-income settings [22] through enabling research to be conducted in a way that is respectful to individuals and communities and social value to be maximized [23]. Towards these aims, community consultation, as a form of community engagement, aims to include 'community voices’ in planning research, including research questions and activities. At the same time, there is unresolved debate on how consultation should be undertaken, including how a community should be defined, who might reasonably represent a community, and how their voices might be listened to and taken into account in practice [24-26]. The consultation methods described in this study used an information-sharing, deliberative approach. Although it is not within the scope of this paper to assess the contribution of this method to the literature on community engagement, we aim to illustrate this contribution in supporting policy decision making in research.

## Methods

### Study site

A detailed description of the KEMRI Wellcome Trust research programme and its setting in Kilifi on the coast of Kenya has been given elsewhere [27]. In summary, the county includes rural and semi-urban populations of around 1 million; subsistence farming is the primary livelihood and between 55% and 65% households live below a poverty line defined in relation to the costs of meeting basic needs [28]. The study was conducted within the population of 260, 000 people resident within the programme’s Health and Demographic Surveillance System (KHDSS) that accounts for around 80% of admissions to the County Referral Hospital [29]. The main population group are Mijikenda [30]; 47% describe Christianity, 13% Islam and 24% traditional beliefs as their faith system. During community engagement planning surveys in 2005, 45% adults reported inability to read a newspaper or letter, although free primary school education was introduced nationally in 2003.

In the research setting for this paper in Kenya, there is a close collaboration between researchers at the KEMRI Wellcome Trust Research programme and government health providers at Kilifi County Referral Hospital that includes the provision of a dedicated weekly clinic for people affected by SC disease, part of wider systematic long-term research support provided to the community through the County Health Management Team [31]. Given this availability of care, researchers in Kilifi generally disclose study-generated findings on SC disease to affected families, but not on SC trait [32]. At the same time, challenges have been experienced with low use of the SC clinic and in providing the resources needed to support disclosure, particularly for large scale studies. As reported elsewhere [27] a particular challenge in research-based SC disease screening in healthy children was the likelihood of 'diagnostic misconceptions’, where the test is seen as health check for the benefit of the individual, similar to the more commonly described 'therapeutic misconception’ of research [33].

### Study population, sampling and data collection

63 residents participated in this study, detailed in Table 1. *A priori* purposive sampling aimed to explore diversity within the population, drawing on features of role, gender and geographic residence (rural and urban). Participants included: i) Residents working full time within the research programme (n=20), including Community Facilitators, Field Workers (staff supporting studies through undertaking informed consent processes, interviews and some sample-taking), Data Entry Clerks and a Scientist in training; ii) District Health Managers (n=4); iii) Administrative leaders, Chiefs and Assistant Chiefs (n=18); iv) KEMRI Community Representatives (KCRs) (n=18), who are 'typical’ residents selected by local communities to support consultation on research-related issues through regular or *ad hoc* meetings with community liaison staff and/or researchers ; and v) Mothers of affected children (n=3), not belonging to the above groups. All groups included people of different ages, religions and educational status.

**Table 1:** Summary of participant information

**Role**	**Total number**	**Gender M**:**F**	**Education (years) range and median**	**Religion**^**a**^	**SC disease history**
*Staff*: *Community facilitators*	5	4:1	12-16y 14y	4C/1M	No direct history
*Staff*: *Field workers*	12	10:2	12-14y 12y	10C/2M	1 field worker - sister has 2 children with SCD
					1 field worker - 2 affected children, 2 further children died SCD
*Staff*: *Others*^*b*^	3	0:3	12-16y	3C	1 data entry clerk - carrier, 2 brothers with SCD, 1 died; carrier child
*Health managers*	4	3:1	16-18y 16y	4C	No direct history
*Chiefs*/ *assistant chiefs*	18	16:2	7-14y 12y	17C/1M	No direct history
*KCRs*: *5 chair*/*vice chairs*; *4 secretary*/*vice secretaries*; *9 members*	18	9:9	3-16y 8y	14C/4M	1 KCR rural area – 1 child with SCD
*Community members*: *affected mothers*	3	0:3	6-12y 6y	3C	2 with 2 affected children; 1 with 1 affected child

^a^C= Christian; M = Muslim.

^b^Two data entry clerks and one junior scientist.

Participants were invited to attend one or two small group discussions (3 to 6 people) to support an informed deliberative process to assess elements of good practice for information sharing on SC disease and trait [34-36] across two stages. Stage one and two discussions were held one week apart at local venues near to participants’ homes or, for staff members, at the research centre; and using the participants’ language of choice (English, Kiswahili or local dialect). The first stage, based on participatory processes, aimed to share information on:

•Prevalence, health implications and forms of management for SC disease;

•SC disease inheritance, particularly transmission across generations by healthy people with SC trait;

•Risks of future children being affected where both parents have SC trait.

The second stage built on understandings shared in the first to explore perceptions of the importance of sharing SC disease and SC trait findings, using two scenarios addressing these different forms of the condition. Stage two discussions aimed to:

•Describe the perspectives of all participants as far as possible;

•Explore reasoning using non-judgmental probes to support reflection, including any emerging morally relevant issues;

•Encourage expression of diversity of views;

•Pay attention to the voices of the most vulnerable within the population, taken here as parents and families of children with SC disease.

### Data management and analysis

Field notes were made during and immediately after meetings. Discussions were recorded, transcribed and translated into English, including a total of 48.5 hours of recordings. All translations were undertaken by note takers present at the meetings, experienced staff in the social science research group with fluency in local languages and English, and checked by FK. Debriefings were held within the study team after each discussion, and findings used to inform the on-going development of the interview guide.

Data were managed using Microsoft Word applications, anonymised through coded identities. The analysis used a modified form of framework analysis [37]. In this study, following in-depth reading of transcripts, we developed a series of analysis charts to capture individual and group level data under themes related to views on the importance of sharing information on SC disease and SC trait information, and underlying reasoning. Charts reflected the flow of debates including changing views to identify individual positions emerging from deliberative discussions. Some themes used in charting were derived from the underlying research questions, based on probes used in discussions. These included perceptions of the likely influence of information sharing on health seeking practices, family relationships and the interests and rights of different stakeholders, and the responsibilities of researchers. Themes were also identified from issues emerging during discussions, including common underlying values, such as openness, truth telling and family stability, and the conflation of SC disease with HIV/AIDS. Analysis was primarily conducted by VM, with support from the other authors, including cross-checking and discussions around coding of data within analysis charts. The varied backgrounds of the authors, including bioethics, social science, public health and community engagement were drawn upon to inform the analysis. VM, SM and FK have lived and worked in the research programme in Kilifi for more than 15 years.

### Ethical review

The study was approved by the Scientific Steering and Ethical Review Committees in Kenya, and the OXTREC committee at Oxford University. Approval included verbal consent for participation, which was given by all participants. The paper is published with the permission of the director, KEMRI.

## Results

In this section, the first two sub-sections describe residents’ views on whether, why and how study-generated information on SC disease and SC trait should be shared with the parents of affected children. The remaining sub-sections describe emerging findings on the influence of context and underlying values.

### Sharing information on sickle cell disease

The findings of this study confirmed those of earlier research based on the experiences of directly affected families [32] of low levels of biomedical awareness of SC disease in the community, and sometimes extreme levels of distress experienced by some families in struggling to understand what was happening to their child, and how best to help them. Confusion was often compounded by the nature of early symptoms of SC disease, being typically fleeting and varied, including episodes of severe unexplained pain and crying, painful swelling of the hands, feet or limbs, yellowness of eyes, fever and abdominal swelling. From this and the earlier study, the main forms of harm of 'not knowing’ about SC disease were, therefore, seen as:

•The exacerbation of ill health and emotional distress for the affected child where relatively ineffective treatment was used, including traditional or faith-based forms of healing;

•Emotional and economic costs to the family associated with looking after the child, including loss of income-generating activities for mothers;

•Forms of blame and conflict within families, particularly affecting mothers blamed for causing the condition in their child. An underlying feature was the tendency for fathers to deny responsibility for ill health in their children, instead placing blame on mothers. Maternal blaming could take the form of fault seen through direct mother-child inheritance, curses or bewitchment; or through claims of a wife’s sexual unfaithfulness and denial of paternity.

#### Reasons for sharing SC disease information: Limiting harms of 'not knowing’

Against this background, participants in this study saw sharing SC disease information during research as of central importance in limiting future serious and avoidable harms for affected children and their families, by optimising care-seeking and reducing the impact of existing harmful 'misconceptions’, rumours and suspicions. In sharing information, participants saw the need to give a full and convincing explanation of the cause of SC disease, in addition to advice on management. Given risks of gendered blaming in families, and a commonly reported belief that effective biomedical care should be curative, many participants particularly emphasised the importance of sharing information on the lifelong nature of the condition and its inheritance from both sides of the family over many generations:

“If the doctor does not explain [the cause and lifelong nature of SC disease] in the end they might feel 'what are we trying to do, and the child is not getting better? The doctor did not tell us anything except always keep going [to clinic] if they don’t know they will stop going in the end instead they will do other things [alternative treatments] while the child continues suffering.” (Female KCR with a child with SC disease, KCR02/P2)

“When I went to the doctor, the child was tested and…we were told that there was a condition from the father and me that caused the child to get that thing. So when we came back home, other people were saying that it was witchcraft…but, the two of us, we knew because the doctor had explained to us, and so we were not worried. So when the parents get information, it removes the fear” (KCR mother of an affected child, KCR02P2)

Some participants, in this and the earlier study [32], described a specific risk that SC disease would be confused with HIV/AIDS, prompted by the need for long term treatment and the typical slim build of people affected by both conditions. In this case, sharing information on the latter would help to reduce confusion:

“When they understand [about SC disease], they can be able to remove that fear from their hearts because some may misunderstand it, they take it this is a disease like HIV/AIDS” (KCR01/P6)

These positive effects of information sharing were seen for the families of affected children included in research. They were also seen as likely to be helpful for other affected family members, including those where the condition was currently unrecognised. In addition, residents felt information was likely to more widely shared, given its perceived importance, and that positive effects would reach others in the community similarly affected.

#### Reasons not to share SC disease information: The risks of generating harm

Whilst there was broad agreement that information should be shared, a number of arguments were proposed against this, and for caution. First, explaining the occurrence of an inherited condition in the family might generate high, and sometimes unwarranted, levels of worry and hopelessness:

“So I think it [sharing information] is important though I have said it’s sensitive because…once you are told that, then there is that feeling that we are all sick, because it is inherited, and so we are all going to have sick children, you know, there is that traumatising effect the parent might get” (male community facilitator, IDI09P1).

Anxiety about the condition was also seen as potentially undermining the parents’ emotional relationship with their affected child and with each other. At an extreme, anxiety about the risks to future children was seen as prompting parents to consider separation:

“Okay now, for me, I can say that there will be many separations… when it’s explained to them in detail the way this is inherited…they can separate because they will not want to have another child with a problem like that.” (Female KCR, KCR01/P2)

Parental separation was seen as a particularly serious outcome for mothers who lacked independent economic resources, as would be likely for many in rural Kilifi. In this traditionally patrilineal culture [38], mothers without independent resources would be likely to have to move back to their maternal homes where economic and social support might not be forthcoming [32]. The position of a chronically sick child accompanying her mother to the maternal home was seen as particularly fragile, given the costs of health care. All the mothers of children with SC disease in this consultation were greatly concerned about the impact of parental separation on a mother and child. At the same time, a few residents saw a potential benefit of separation where both parents had independent livelihoods:

“…and if the worst comes to the worst and the family breaks up…if this man decides to marry another wife maybe he can get one who is not a carrier, and the lady can also get a man who is not a carrier, and they start very fresh lives.” (Female field worker, IDI07/P4)

Although explaining parents’ individual genetic roles was seen as important to address paternal denial of responsibility, paradoxically, this information also carried a risk of generating or increasing gendered blame in families. This risk was sometimes very strongly articulated, and seen as occurring where fathers continued to deny their roles, if information was misinterpreted, or where information generated doubts about paternity of the child. As before, the consequences of maternal blame were seen as potentially serious for the mother and child. This point remained controversial across all groups, although most perceived the risks of gendered blaming to be greater *in the absence* of a good explanation about the roles of both parents than with this. All the women with an affected child in these discussions supported this view:

“Because even if you don’t tell them, if they are going to divorce, they will divorce…[for example thinking] 'this woman is evil, every time she gives birth it’s a sick child, maybe they [family] have evil spirits, so no! You go to your home, you can even take the child, I don’t need the child!’ But…if they will have been told, they will understand.” (KCR with an affected child, KCR01/P5)

### Sharing information on sickle cell trait

As for SC disease, many residents felt that study-generated information on SC trait should be shared with families, but with greater differences and shifting of opinions as new points of view were put forwards and considered. Reasons not to share information on SC trait were more commonly and strongly voiced than any raised in support of withholding any type of information on SC disease. A major influence was recognition of the current lack of public access to SC disease screening for healthy individuals, as described in the following sections.

#### Reasons to share information on SC trait

There was almost complete agreement that individual knowledge of SC trait would be very important to allow families’ choice in reducing a child’s future reproductive risks if screening for this condition was widely available. In the absence of this service, many felt sharing SC trait information would be less valuable. However, it was often seen - sometimes strongly - that information would help families to be prepared for this eventuality, including understanding how to seek effective care:

“At least, s/he will be prepared [*Kiswahili*: *amejiset*]. At the time s/he comes to marry and if s/he has a child like this, s/he will remember 'eeh I was told, in the past I was told.’” (Male KCR, KCR01/P4)

This benefit to families was often linked to a perceived public health importance of creating wider awareness of this genetic risk:

“He [the child] is fine, yes, he is fine, but… we have to enlighten the parents that this problem may come up in future…if this information is not known and the condition ends up affecting those born in future, how will this problem be solved? So I think they [parents] need to be given the full information.” (KCR, KCR02/P4)

In addition, some saw that sharing information on SC trait could empower families to create a demand for more and better SC disease services, both at a policy and an individual level.

#### Reasons not to share information on SC trait

As for SC disease, disadvantages were related to anxieties thought likely to occur, including through misinterpretations. The key difference was that, for SC trait, these worries were seen as being generated without 'good reason’, given the inability to manage future reproductive risks by screening a partner. At times, this view was strongly held, including as a right 'not to know’:

“I mean you will explain as well as you can, but later you will leave that particular person worried. When he [the child] comes to marry later, he will say that it would have been better if I didn’t agree for him to be tested…” (KCR, KCR02/P6)

“I just don’t think it’s worth knowing…if there is no structure in place to say test the other person … I would rather just stay the way I am [not be told].” (Community facilitator, IDI09/P4)

Given the anxieties involved, some felt that SC trait screening should target young adults, including before marriage, but not infants or children. Others disagreed, seeing that young people would be particularly vulnerable to emotional upset from genetic screening:

“When you tell him directly that you have this then he will be thinking a lot, and even consider committing suicide because even when I get married things won’t be good.” (Male KCR, KCR03/P4)

Concerns about unnecessary worry were compounded by views that SC trait information could easily be misinterpreted as having a direct impact on the child’s health, either in the short or long term:

“…so they will remember all through that my child is sick, not my child has a condition which needs a person to make a good decision during marriage, but they will just remember my child is sick…that thing cannot move out of their mind” (Male community facilitator, IDI09/P1)

One feature of the risk of misinterpreting the term 'carrier’ was the common use of this term in referring to SC trait and carrier status in HIV/AIDS, leading to conflation between these types of 'carriers’ or to the conditions themselves. A further local form of medicalisation of SC trait was described as the potential for anxiety to lead some people to seek traditional treatment to 'remove’ the risk.

These views on the potential risks of sharing SC trait information were not universally held. Some felt that anxiety about SC trait was unlikely to be important in practice; the experience of normal health in affected children would lead parents to accept the condition as non-harmful or forget the information in time. Acceptance would be helped by parents’ reflection on their own health, since at least one parent must have SC trait; and the much greater priority likely to be given to hardships confronting many people in the community on a daily basis.

A different and particularly key challenge in sharing SC trait information - raised by relatively few residents, but strongly influencing views in their groups - was the low likelihood that parents would be able to accurately and sensitively pass SC trait information on to their children in the future, particularly given that this might happen in 10 or more years’ time. A chief compared this situation to HIV/AIDS control policy:

“Me I still disagree, they should not be told…we have a programme in my area on HIV/AIDs infected children. When a child reaches seven or eight years you are supposed to disclose the news to the child, but there is no parent who discloses the news to the child…So it will be the same thing with sickle cell…” (Chiefs 01/P3)

Within these discussions, others continued to feel that parents were the right people to pass this sensitive information on to children:

“They can help to prepare and explain this to the child over time. They can also explain that being a carrier is not an illness, particularly since they themselves are also carriers.” (IDI01)

Finally, some individuals in some groups saw risks of stigmatisation for children with SC trait, including difficulty in finding marriage partners in future. Countering, others saw that children and families had the option to keep carrier information confidential; and that carrier knowledge and greater openness could work towards reducing stigmatisation. The latter point was made particularly strongly by all mothers of affected children. As a community facilitator said:

“I think if you borrow a leaf from what HIV/AIDS campaign have been doing, the kind of education which is being provided is fighting that stigma, yeah, it has spread very much…” (IDI09/P3)

Additionally, in relation to risks of gendered forms of blame and stigma in affected families, knowledge and acceptance of carrier status *before marriage* was seen to support mutual trust and help parents take care of an affected child in the future, if this were an outcome.

### The influence of context on perceptions of outcomes of information sharing

Diversity of opinion featured throughout the discussions described so far, to a large extent related to recognition of multiple influences on the way information sharing might work out for different families. This context-dependent feature of the impact of sharing genetic information has been described in the literature as situated processes of co-construction [39], illustrated here through the interdependence seen for structural features of context, family dynamics and the nature of information shared.

#### Macro level influences

##### Cultural and socioeconomic circumstances

Economic status was seen to have a particularly important influence on the benefits of sharing information, particularly for SC disease. For either parent, having independent economic resources was thought to reduce the risks of being 'abandoned’, but this was particularly important for mothers. For example, chiefs described that mothers able to bring an income into the family would be more likely to influence family decision making, and less vulnerable to blaming by their husbands. Women in the urban KCR group, particularly individuals with high levels of education, talked positively of their ability to manage their lives even if separation from a partner occurred. Overall, mothers without an independent livelihood were seen as vulnerable to harm in situations of 'not knowing’ and 'knowing’ about SC disease. Illustratively, a member of the District Health Management Team spoke of this as a 'pathetic situation’.

The cultural practice of polygamy also provided an influence in the way some families might resolve anxieties about future reproductive risks in Islamic and traditional faiths where the practice is supported and amongst followers of other religions. In these situations, the existence of strong emotional bonds within a family was seen as a reason to choose polygamy in preference to separation.

Many residents also raised a role for formal education as an influence on the benefits of sharing information, often based on an assumption that greater exposure to schooling would reduce risks of misunderstanding genetic information, a controversial point:

“The highly educated people are the most difficult to explain to, but the ordinary people will listen and think, because education is different to intelligence.” (Male KCR, KCR01/P6)

In general, increased schooling in women was generally related to greater potential for economic independence, and reduced vulnerability to blame and the harms of family breakup, serving as a marker for valued forms of development:

“For one, I think we are moving from the older days…I think to be sincere more people have gone to school, they can understand even the genetic part of this information as opposed to long time ago, and I think people who blame maybe the wife for the genetic makeup - though there’s still a gap - but I don’t think that’s so big” (Community facilitator, IDI09/P1).

#### Micro level influences

##### Individuals and family relationships

Alongside descriptions of the influence of culture and socioeconomic factors, residents agreed that there was considerable variation within families in the way that individuals relate to each other, seen as a very important influence on the outcome of family disputes [40]. The most commonly described and important positive concepts were those of trust and emotional commitment, with jealousy as a counter. The likelihood of fathers accepting their genetic role in SC disease, including having SC trait and being the parent of an affected child, was seen as dependent on the emotional bonds and levels of trust already in place in the family. Where these existed, paternal denial, gendered blaming and separation were much less likely.

To some extent, observed patterns of SC disease within the family were thought to play into these interpretations, since a common response to recognition of an unknown illness would be to scrutinise extended families on both sides over several generations to see whether any patterns could be established suggesting the origins of the problem [32]. Similar practices have been described for genetic conditions within families in high-income country settings [41]. However, there was little agreement on the implications of particular patterns. Explaining reproductive risks to young couples with one affected child might increase the risk of family breakup, given that no other children would be involved; but parents in this situation might not take a theoretical future reproductive risk seriously. It seems likely that patterns may be less important than other contextual features, particularly the attitudes of individual parents and the existence of trust, as described previously.

### Emerging underlying values

While differences of opinion were often based on the influence of macro and micro level features in different families, tension was also associated with controversy in the way two important underlying values in these discussions were prioritised. A generally dominant value was the protection of vulnerable children and family stability, seen as inter-dependent, and described here as a value of 'family interests’. In addition, openness – often as a form of empowerment – was highly valued by many participants. While these values are likely to have gained prominence from the focus of these discussions on children affected by a serious genetic condition, they played an important role in strengthening convictions and concerns through their alignment or tension, respectively, within debates.

#### Family interests

##### The interests of vulnerable children, their mothers and families

In weighing up the potential benefits and disadvantages of sharing SC information described so far, many residents placed the interests of children with SC disease and their mothers, linked to stability and harmony in families, as their main priority. A lesser degree of vulnerability for children with SC trait was generally reflected in a less heated defence of their individual interests; instead more controversy drew on the public health importance of SC trait.

In the literature, the concept of family is complex. A distinction has been made between two broad meanings: the household or 'aggregate or group of actual members who are closely associated by living arrangement or by commitment, for better or worse’; and the family in abstract, including 'the family line…whose boundaries extend over space and time’ [42](p35). The value of family stability in these discussions seems to relate to the former, and often referenced structural economic arrangements focused on the parents, affected child, siblings and other dependent family members. At the same time, intrinsic values of the 'actual family’ and its stabilising role in wider family society were also described:

“What I’m saying is…you cannot make this conflict rise in that home, because by all, either right or wrong…you have to make them live together.” (Chiefs 02/P2)

#### Openness and empowerment

A positive idea of the value of openness underpinned many views on the benefits of sharing SC information, as a form of empowerment through knowledge for individuals and the wider community. For example, individual families would be able to take more control over their lives (impacting care of an affected child), experience less blame and stigmatisation, and be better able to manage future reproductive risks:

“Information is power…so if they get the knowledge [about SC disease], they will continue managing the kids very well, ignoring the others, whatever the other people will be saying.” (Male field worker, IDI07/P3)

At a community level, openness about SC disease and SC trait was also linked to the potential for advocacy and reducing stigmatisation, the latter often linked to HIV/AIDS control policy:

“Ok…back in 1984, 1982, when the first AIDs cases were discovered.... I think in this same case [of SC disease]…many people don’t know or don’t understand about it…Now we are living with HIV patients.... it’s no longer an issue as it used to be. [For SC disease] as time goes by…a few members of the family will accept, and then later a few members of the village accept, and then later on a few members of the location accept…” (IDI07/P3)

Similarly, a field worker told the story of a mother whose child had been found to have SC disease during research, and whose attitude had changed from an initial one of denial and anger to one of positively seeking to influence community knowledge and attitudes by becoming a local advocate for SC disease in her village. The suggestion that knowledge could empower affected families or communities to lobby for better SC disease services in the future also reflects a community level benefit. At the scale of a specific project, this may be difficult to imagine, but patient advocacy and public pressure have in fact been the driving force for SC disease programmes in many parts of Africa where these exist, in the form of charitable foundations [18].

Finally, several residents described a potential negative impact of *a lack* of openness about SC trait information, reflecting on policies at that time for the genomics study in Kilifi. The risk was of loss of trust in researchers (and the research institution) by individuals or the wider community if it became known that SC trait information had been withheld. Examples of ways this might happen included future SC disease screening with a conflicting result, or the birth of an affected child in a future generation of the family:

“Maybe we may just say that we don’t want to displease the parents, but then after that, yes, the kid will be a carrier and nothing will change that…when he or she marries another carrier, ok, 'we were told that the kid was negative, how did this come about? Or was there something which was being hidden as to..?’ Ok, it will bring some question marks.” (Field worker, IDI06/P1)

## Discussion

In this study, we aimed to explore informed views of a range of residents in Kilifi County in Kenya on how researchers should manage information generated on SC disease and trait during studies as a form of community consultation. We sought to understand views on what information should be shared and how, and with what potential benefits, risks and other ethical implications, to contribute to the development of policy. In this discussion, we highlight key findings and their implications, and lessons learned for methodology in community consultation in this and similar settings.

### Sharing study-generated information on SC disease and SC trait

Overall, the consultation indicated a high ethical importance of sharing study-generated information on SC disease in children with their families. Community perceptions of benefit show the case for disclosure as compelling based on strong and agreed views on the likelihood of limiting probable and severe harms for many participant children, their mothers and families. This conclusion is underlined by research guidelines on the disclosure of genetic findings that reference the importance of taking account of clinical and social benefits (or utility) of sharing information [13].

The study also illustrated the sensitivities involved in sharing SC disease information, with risks of generating unnecessarily high levels of anxiety in families and putting sometimes extreme strain on parental relationships. Any resulting parental separation was seen as engendering very severe hardship for some mothers and affected children. In addition, an aim of sharing information on parental roles in SC disease to reduce risks of paternal denial of responsibility for their child’s condition could paradoxically increase these risks, depending on the influence of existing relationships, socioeconomic context and the way in which information is shared. A specific challenge was the risk of generating paternal requests for individual screening to 'test’ for paternity. Community views on what researchers’ responsibilities might be in this situation were mixed, but we have argued [43] that researchers should not test parents since harms likely to be caused by showing misattributed paternity, even if in few cases, outweigh responsibilities to counter paternal denial in this way.

In the case of SC trait, where the perceived harms were seen as less immediate and severe, there was also greater disagreement on the benefits of sharing information. Disagreement and ambivalence were strongly underpinned by the lack of public access to screening for SC trait in healthy adults in Kenya, and by a series of concerns, including: parents’ ability and willingness to pass on genetic information to their children in future; the psychological effect of this information on affected individuals; and risks of stigmatisation, including where the word 'carrier’ was conflated with the use of this term in HIV/AIDS. In this way, a few participants supported a right not to know about carrier status in these circumstances. At the same time, different forms of benefit from sharing SC trait information were seen, including the ability to create wider public awareness of SCD, such that public advocacy could apply pressure for wider screening services to be set up. A central benefit seen was that of 'preparedness’, with the potential to limit future harms through better understanding of the condition. Relatedly, awareness of SC trait status within a partnership before marriage was seen to have the potential to limit risks of parental separation and gendered blame if an affected child was later conceived. In any case, participants were concerned that failing to share SC trait information, where this was known by researchers, could undermine relationships between residents and researchers where this was seen as 'withholding’ important information. One implication of this diversity of opinion is that the concept of healthy carrier status in SC disease - and for the potential for this to be disclosed by research - should be shared during the consent process, and participants given a choice about accessing information on SC trait where this is seen as helpful. A similar approach has been taken for carrier findings during newborn SC disease screening programmes in Ghana [9], and is recommended by the European Society of Human Genetics [44].

An important challenge to views that study-generated SC disease and trait information should be shared that was not considered in depth during the consultation comes from considering the resources, and appropriateness, of researchers taking responsibility for supporting counselling and clinical services for a lifelong health condition of public health importance. Apart from arguments about resource prioritisation in research, research funding cycles are often incompatible with providing lifelong services; and researchers taking on this role may undermine that of Ministry of Health partners [15]. Resource arguments are particularly challenging for disclosure of SC trait findings, where the perceived benefits are less urgent and certain than for SC disease, and resources requirements to validate tests and provide counselling are greater, given its higher prevalence (18% SC trait vs. 1% SC disease prevalence in infants in Kilifi). In this way, the findings highlight the challenge, described in the literature on disclosing genetic findings, of balancing the benefits of disclosure to individual study participants against the resources needed to support realisation of these benefits [15,45], in this case through ensuring provision of SC services, including clinical care and counselling. On this basis, while the absence of effective public services may seem to suggest limits to researchers’ responsibilities for disclosure in some instances, our findings highlight the moral challenges of failing to share study-generated information on SC disease. Rather, the ethical importance of limiting harm in this situation, together with the public health nature of SC disease, underlines the importance of researchers working in prior partnerships with government health authorities to ensure that - as far as possible - disclosure and services support the long term interests of study participants.

### Learning about community consultation

Community consultation is widely recognised as important in supporting ethical practice in many types of research, particularly to take account of potentially conflicting principles emerging from theory or practice [46,47]. There is less published experience or guidance on approaches to community consultation, including on ways of consulting on technical aspects of research that are not necessarily familiar to potential participants [25] . Our experiences suggest that building information-sharing activities into the consultation was essential to the complexity of discussions, given low awareness in the community of many ethically important biomedical aspects of SC disease and its inheritance. In addition, using deliberative methods - including re-visiting issues over time - facilitated a strong and reflective engagement, and generated diverse and detailed accounts of the views and values of residents.

Diversity was largely generated in two ways: firstly, by participants recognising inter-related individual and contextual influences on the likely impact of information sharing; and, secondly, by participants’ different ways of prioritising underlying values. The depth of exploration gave insights into the central importance attached to family interests and policies of openness; alignment of these values often underpinned strength of opinion and levels of agreement. Similarly, tension between these values or their prioritisation in specific situations lay behind much disagreement. The ethical implications of both forms of diversity suggests that community consultation should be based on carefully accounting for difference, including in the voices listened to, the sharing of information to support debate and the use of methods that explore reasoning and reflection over time, without resort to consensus building.

At the same time, further research on these and other methods for consultation are important to strengthen understanding of the potential contribution of these findings to policy, including how typical these 'community voices’ are, whether and how wider community accountability should be sought and whether views based on the assimilation of relatively new understandings might importantly continue to develop over time. In particular, an iterative approach involving feeding back of the findings to community and other national research stakeholders in Kenya would be important in developing wider policy [48].

## Conclusions

This study has shown the high ethical importance and the sensitivities of sharing study-generated information on SC disease in children in Kilifi and other similar settings, including the potential to limit harms that may otherwise be very severe for affected children and their families. In this consultation, arguments for sharing SC trait information created greater controversy, and were less compelling in terms of the nature and probability of harms potentially limited. The extent of diversity in views on sharing carrier status suggests that disclosure policies that support individual choice would importantly maximize the possibility of individual benefit. The public health nature of SC disease and the ethical importance of limiting harm in these situations emphasise the importance of researchers whose studies include SC screening working in prior partnerships with government health authorities to ensure that - as far as possible - disclosure and services support the long term interests of study participants.

Theoretical questions on approaches to normative analysis of empirical findings from community consultation on health research practice, and the role of *in situ* empirical debates, remain far-reaching [49] and have not been addressed in this paper. In fact, there remains a gap in understanding which data collection and analysis methods would be most effective in community consultation on health research, particularly for technical and unfamiliar aspects of studies [25]. Within these limitations we conclude that approaches using deliberative discussion and drawing on shared information, normative reflection and an exploration of the potential for difference can importantly enrich ethical debates on good practice.

## Competing interests

The authors declared that they have no competing interests.

## Authors’ contributions

All authors contributed to the design of the study, analysis of findings and writing the manuscript. VM and FK conducted the community consultation and collected the data. VM and FK were primarily responsible for data collection. VM was primarily responsible for analysis, drafted the first version of the manuscript and edited subsequent versions. All authors read and approved the final manuscript.

## Authors’ information

VM, FK, TNW and SM have worked at the KEMRI Wellcome Trust Research Programme and been resident in Kilifi for more than 15 years. VM is a senior researcher in the KEMRI Wellcome Trust research programme and University Research Lecturer at Oxford University, with a background in medical and social science. FK is a community facilitator at the KEMRI Wellcome Trust research programme with a master’s degree in public health. RF is a social scientist, Professor of Public Health and Head of the Department of Public Health at Oxford University. TNW is a Senior Research Fellow and Professor of Medicine at the KEMRI Wellcome Trust research programme and Imperial College, London; his research focuses on haemoglobinopathies in Africa. MP is the director of the Ethox Centre at Oxford University. SM is a social scientist and Wellcome Trust Research Fellow at the KEMRI Wellcome Trust research programme and Oxford University.

## Pre-publication history

The pre-publication history for this paper can be accessed here:

http://www.biomedcentral.com/1472-6939/14/41/prepub
